# Meta-analysis of diagnostic accuracy of neutrophil CD64 for neonatal sepsis

**DOI:** 10.1186/s13052-016-0268-1

**Published:** 2016-06-07

**Authors:** Jing Shi, Jun Tang, Dapeng Chen

**Affiliations:** Department of Pediatrics, West China Second University Hospital, Sichuan University, No 20 Section 3 South Renming Road, Chengdu, Sichuan Province 610041 People’s Republic of China

**Keywords:** Neutrophil CD64, Neonatal sepsis, Diagnosis, Meta-analysis

## Abstract

**Background:**

The aim of this study was to systematically evaluate the diagnostic performance of nCD64 for neonatal sepsis.

**Methods:**

Computer retrieval was conducted for the databases of PubMed, Embase, and Springer databases up to March 18, 2015 to select the relevant studies on nCD64 and neonatal sepsis. Sensitivity, specificity, positive likelihood ratio (PLR), negative likelihood ratio (NLR), diagnostic odds ratio (DOR), and 95 % confidence intervals (CI) for diagnostic efficiency of nCD64 were pooled. In addition, the summary receiver operating characteristic (SROC) curve was also conducted based on the sensitivity and specificity.

**Results:**

Seventeen studies including 3478 participants were included in this meta-analysis. The overall pooled sensitivity, specificity, PLR, NLR and DOR were 0.77 (95 % CI: 0.74–0.79), 0.74 (95 % CI: 0.72–0.75), 3.58 (95 % CI: 2.85–4.49), 0.29 (95 % CI: 0.22–0.37) and 15.18 (95 % CI: 9.75–23.62), respectively. In addition, the area under the SROC curve (AUC) was 0.8666, and no threshold effect was found based on the Spearman correlation analysis (*P* = 0.616). Besides, subgroup analysis showed higher sensitivity, specificity and AUC in term infants and proven infection group than those in preterm infants and clinical infection group, respectively.

**Conclusions:**

The n CD64 expression alone is not a satisfactory marker for diagnosing neonatal sepsis with relatively low sensitivity, specificity, PLR and NLR, in spite of relatively high SROC area. Therefore, the n CD64 expression used in diagnosis of neonatal sepsis should be treated with caution.

## Background

Neonatal sepsis is one of the important causes of neonatal mortality. Despite the improvement in management of newborn infant, the mortality caused by neonatal sepsis remains high (~10 %) [1]. It is difficult to diagnose neonatal sepsis during early stage because of the nonspecific and variable clinical symptoms. Blood culture is the current golden standard for confirming the neonatal sepsis. However, the results of blood culture could be available within 24–48 h of culture. Usually, the antibiotics would be discontinued if the blood culture results were negative by 48 h [2, 3]. Moreover, the results are negative in cases with meningitis and pneumonia [4]. There is a high false-negative rate of blood culture [5]. Therefore, considering the limitations of blood culture in neonatal sepsis diagnosis, new biomarkers for early and rapid diagnosis of neonatal sepsis should be developed.

Recently, neutrophil CD64 (nCD64) has been reported as a diagnostic marker of neonatal sepsis, because nCD64 expression is stable for 24 h and can be detected rapidly by flow cytometer with minimal blood volumes [6]. However, the diagnostic accuracy of nCD64 remains unclear due to the large range of sensitivity (0.26–0.95) and specificity (0.62–0.97) in different individual studies [7–9]. Although a meta-analysis has been conducted by Jia et al. in 2013 [10], they combined the results of median monocyte/nCD64 ratio with nCD64 expression, which might be a source of heterogeneity. In addition, recently new individual studies [11, 12] on this topic have reported conflicting results with Jia et al. [10]. Thus, there is a need to update the exploration.

In this study, we performed an updated meta-analysis to systematically evaluate the diagnostic performance of nCD64 for neonatal sepsis.

## Methods

Because the data of this manuscript come from the public databases and previous studies, it is not applicable to receive the ethics committee approval or follow the Declaration of Helsinki, and there is no need to get informed consent of patients.

### Search strategy

We systematically searched the PubMed, Embase and Springer databases up to 18 March, 2015 with the following search terms: (septicemia or septicaemia or sepsis or infection) and (neutrophil CD64 or nCD64). We also manually searched the printed articles, and the references of the reviews and the included studies.

### Inclusion and exclusion criteria

The studies were included if they met the following criteria: 1) exploring the diagnostic value of the nCD64 for sepsis; 2) reporting the babies within 28 days of birth; 3) providing the golden standard of blood culture; 4) giving the number of true positive (TP), false positive (FP), true negative (TN) and false negative (FN).

The following studies were excluded: 1) the studies were written in a language other than English; 2) reviews, letters and reports.

### Data extraction and quality assessment

Two investigators independently extracted the following data using a standard form: name of the first author, publication year, study region, diagnostic golden standard, detection method and cut-off value of nCD64, TP, FP, TN and FN. They exchanged the form after filling out the data extraction. Discrepancies were solved by discussing with each other.

The quality of the included studies was assessed by using a 14-item Quality Assessment of Diagnostic Accuracy Studies (QUADAS) list [13]. Each item was descriptively assessed with yes, unclear or no and scored by 1, 0, -1, respectively [14]. Total scores were produced by plus the scores of each item.

### Statistical analysis

The statistical analysis was performed using Meta-disc software (version 1.4) [15]. The sensitivity, specificity, positive likelihood ratio (PLR), negative likelihood ratio (NLR), diagnostic odds ratio (DOR) and 95 % confidence interval (CI) for diagnostic efficiency of nCD64 were pooled. The heterogeneity among studies was evaluated by Cochran *Q* test and *I*^*2*^ statistic [16]. *P* < 0.05 or *I*^*2*^ > 50 % was considered statistically significant and a random effects model was used for pooling the data; otherwise, a fixed effect model was utilized. The summary receiver operating characteristic (SROC) curve was also conducted based on the sensitivity and specificity. The area under the curve (AUC) close to 1 indicated a good diagnostic performance of nCD64 [17]. Threshold effect was assessed using Spearman correlation analysis, and *P* < 0.05 indicated a significant threshold effect [18]. Subgroup analyses based on the diagnosis standard for infection (clinical or proven infection), type of sepsis (early-onset or late-onset), infants (preterm or term) were conducted. Clinical infection means infection suspected on a clinical basis whereas proven infection means culture proven infections with an identified microorganism. In addition, a meta-regression analysis was conducted based on the above variances to explore the sources of heterogeneity.

## Results

### Study selection

**Fig. 1:**
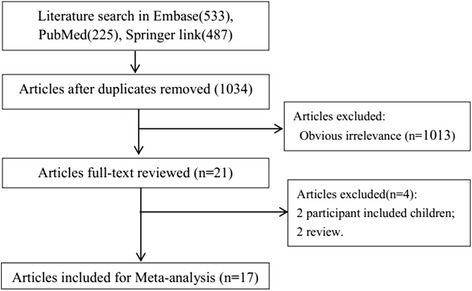
The process of the study selection

The process of the study selection is shown in Fig. 1. We identified 1,245 studies by the initial search (Embase: 533, PubMed: 225, Springer: 487). Firstly, 211 duplicate studies were removed. Then, by reviewing titles and abstracts, 1,013 studies that did not meet the inclusion criteria were ruled out. In addition, 2 reviews and 2 studies including children population were precluded by reading full-texts. Finally, 17 studies [7–9, 11, 12, 19–30] were included in this meta-analysis.

### Characteristics of the included studies

The characteristics of the 17 included studies were listed in Table 1. There were totally 3478 participants involved in this meta-analysis. Nine of the included studies distributed in Asia, 2 in Europe, 5 in America and 1 in Africa. The diagnostic golden standard included clinical test, hematological and biochemical laboratory investigations, and microbiological test-blood culture. The expression of nCD64 was assessed by flow cytometry. As shown in Table 2, the quality of the included studies was relatively high, because most of the total scores ≥ 10.

**Table 1:** Characteristics of included studies

ID	Author	Year	Area	n	Episodes of sepsis	Infected/noninfected	Diagnosis standard	Type of sepsis	Infants	nCD64 analysis	Analysis cut-off	TP	FP	FN	TN
1	Bhandari	2008 [7]	USA	163	293	128/165	Clinical or proven	b	Preterm	FCM	2.30*	90	63	38	102
2	Dilli	2010 [21]	Turkey	109	109	35/74	Clinical or proven	c	Preterm + term	FCM	4.39*	31	11	4	63
3	Genel	2012 [22]	Turkey	119	119	49/70	Clinical or proven	c	Preterm + term	FCM	3.05 MFI	40	16	9	54
4	Groselj-Grenc	2009 [23]	Slovenia	46	46	17/29	Clinical or proven	c	Preterm + term	FCM	1.86*	13	6	4	23
5	Lam	2011 [24]	China	310	310	136/174	Clinical or proven	a	Preterm + term	FCM	6010 $	107	37	29	137
6	Motta	2014 [25]	Italy	129	129	48/81	Clinical or proven	a	Preterm	FCM	2.4*	31	12	17	69
7	Ng	2004 [26]	China	359	359	115/244	Clinical or proven	a	Term	FCM	5500 $	93	46	22	198
8	Ng	2006 [27]	China	298	298	93/205	Clinical or proven	a	Term	FCM	6136 $	73	20	20	185
9	Zeitoun	2010 [30]	USA	98	98	49/49	Clinical or proven	c	Preterm + term	FCM	2.6*	45	14	4	35
10	Du	2014 [11]	China	158	158	88/70	Clinical	a	Preterm	FCM	1010 $	72	21	16	49
11	Elawady	2014 [12]	Egypt	50	50	25/25	Clinical	b	Preterm + term	FCM	46.0	24	0	1	24
12	Layseca-Espinosa	2002 [8]	Mexico	29	29	14/15	Clinical	c	Preterm + term	FCM	#	3	0	11	15
13	Streimish(a)	2014 [29]	USA	684	1156	207/416	Clinical	a	Preterm	FCM	1.63	139	137	68	279
14	Streimish(b)	2014 [29]	USA			204/329	Clinical	b	Preterm	FCM	2.19	159	135	45	194
15	Choo	2012 [20]	Korea	23	23	11/12	Proven	c	Preterm + term	FCM	3.0*	10	2	1	10
16	Elawady	2014 [12]	Egypt	50	50	25/25	Proven	b	Preterm + term	FCM	45.8	24	0	1	25
17	Layseca-Espinosa	2002 [8]	Mexico	34	34	17/17	Proven	c	Preterm + term	FCM	#	5	1	12	16
18	Ng	2002 [9]	China	110	147	37/110	Proven	b	Preterm	FCM	4000 $	35	13	2	97
19	Soni	2013 [28]	India	60	60	24/36	Proven	c	Preterm + term	FCM	2.765*	22	12	2	24
20	Streimish(a)	2012 [19]	USA	649	997	3/577	Proven	a	Preterm	FCM	2.38	3	185	0	392
21	Streimish(b)	2012 [19]	USA			47/370	Proven	b	Preterm	FCM	3.62	35	85	12	285

a, early-onset; b, late-onset; c, early & late-onset; *, CD64 index; FCM, flow cytometric technology; MFI: mean fluorescence intensity; Clinical infection defined as infection suspected on a clinical basis; proven infection defined as culture-proven infection with an identified micro-organism; #, Arithmetic mean + 3 SD of the percentage of CD64 + cells found in normal neonates; $: cAntibody-phycoerythrin molecules bound per cell

**Table 2:** Quality assessment of the included articles

Studies	QUADAS list item													
	1	2	3	4	5	6	7	8	9	10	11	12	13	14
Bhandari, 2008 [7]	+	0	+	+	+	+	+	+	0	0	+	+	0	+
Choo, 2012 [20]	+	-	+	+	+	+	+	+	0	0	+	+	-	+
Dilli, 2010 [21]	+	+	+	+	+	+	+	+	+	0	+	0	+	+
Du, 2014 [11]	+	+	+	+	+	+	+	+	-	+	+	+	0	+
Elawady, 2014 [12]	+	+	+	+	+	+	+	+	+	0	+	0	+	+
Genel, 2012 [22]	+	+	+	+	+	+	+	+	0	0	+	0	+	+
Groselj-Grenc, 2009 [23]	+	0	+	+	+	+	+	+	+	0	+	+	+	+
Lam, 2011 [24]	+	0	+	+	+	+	+	+	+	+	+	+	0	+
Layseca-Espinosa, 2002 [8]	+	0	+	+	+	+	+	-	+	0	+	0	0	+
Motta, 2014 [25]	+	-	+	+	+	+	+	+	+	0	+	0	+	+
Ng, 2002 [9]	-	+	+	+	+	+	+	+	0	0	+	0	+	+
Ng, 2004 [26]	+	+	+	+	+	+	+	+	+	0	+	+	+	+
Ng, 2006 [27]	+	+	+	+	+	+	+	+	+	0	+	+	+	+
Soni, 2013 [28]	+	0	+	+	+	+	+	+	+	0	+	+	+	+
Streimish, 2012 [19]	+	0	+	+	+	+	+	+	0	0	+	+	0	+
Streimish, 2014 [29]	+	0	+	+	+	+	+	+	0	0	+	+	0	+
Zeitoun, 2010 [30]	+	+	+	+	+	+	+	+	0	0	+	0	0	+

*Abbreviation*: *QUADAS* Quality Assessment of Diagnostic Accuracy Studies. +: YES; -: NO; 0: not clear

### Pooled analysis

**Fig. 2:**
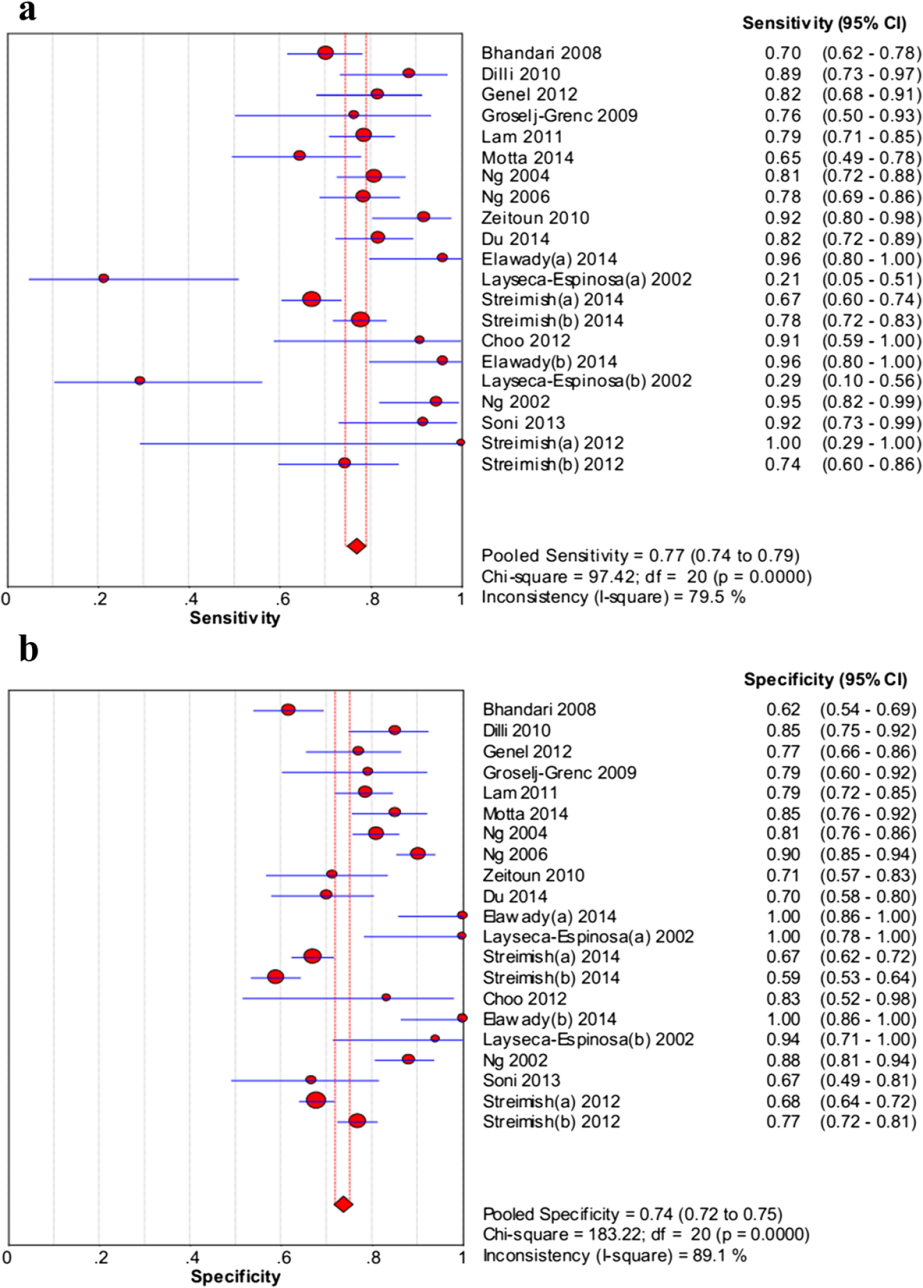
The forest plots of sensitivity (**a**) and specificity (**b**) of neutrophil CD64 for neonatal sepsis diagnosis

**Fig. 3:**
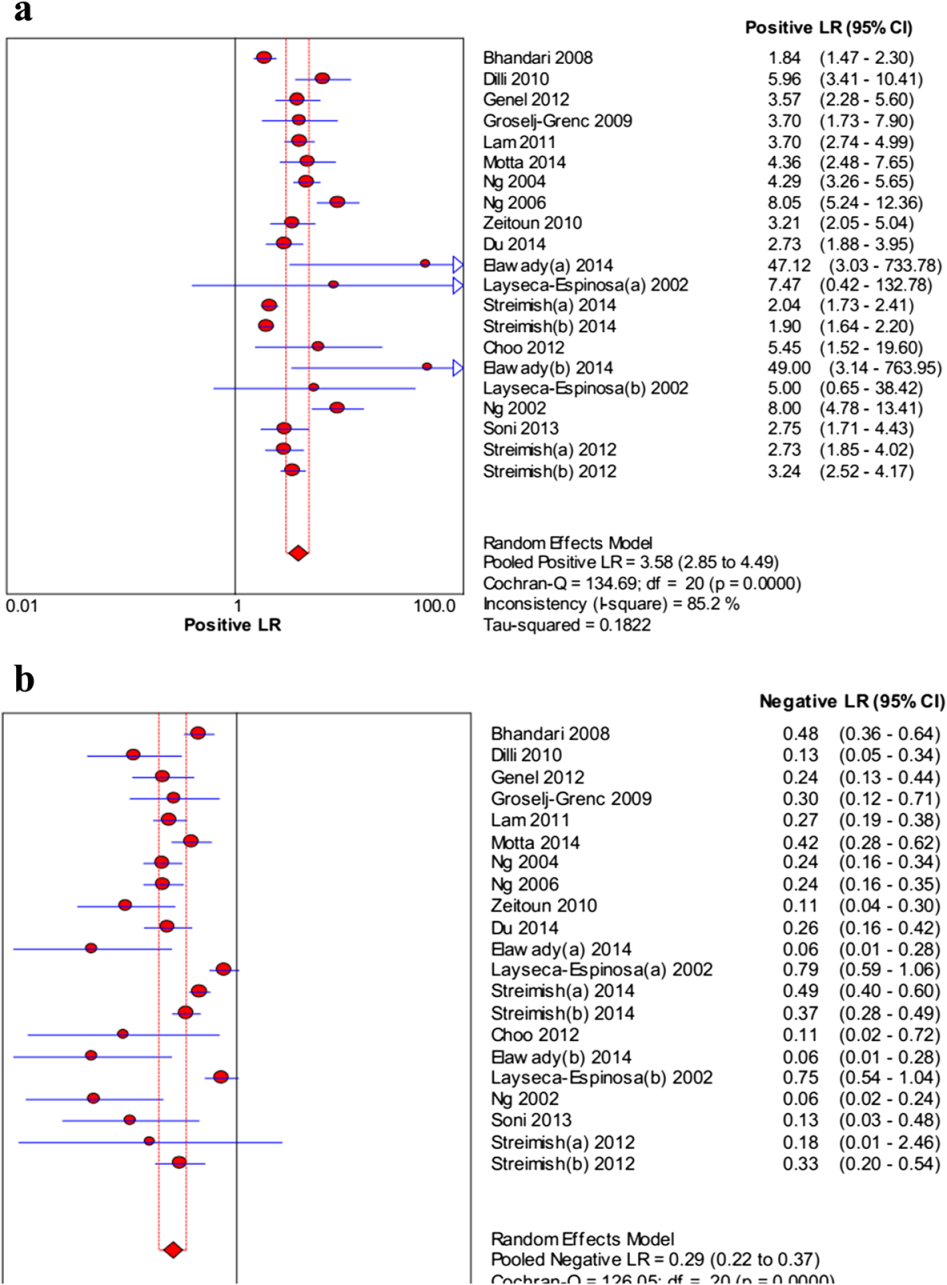
The forest plots of positive likelihood ratio (**a**) and negative likelihood ratio (**b**) of neutrophil CD64 for neonatal sepsis diagnosis

**Fig. 4:**
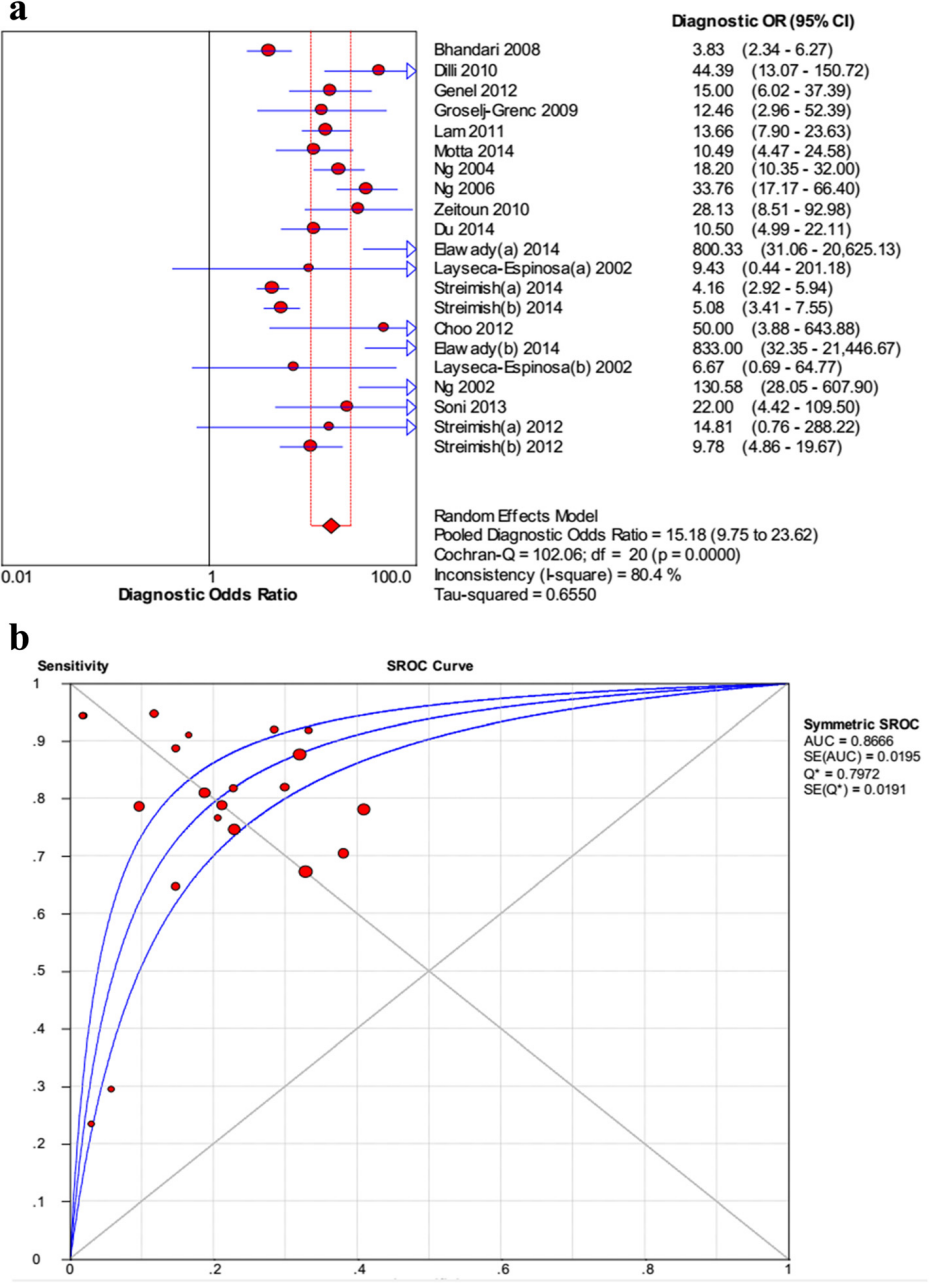
The diagnostic odds ratio (DOR) (**a**) and the summary receiver operating characteristic (SROC) (**b**) curve

As shown in Fig. 2, the pooled sensitivity and specificity were 0.77 (95 % CI: 0.74–0.79) and 0.74 (95 % CI: 0.72–0.75), respectively. The pooled PLR and NLR were 3.58 (95 % CI: 2.85–4.49) and 0.29 (95 % CI: 0.22–0.37), respectively (Fig. 3). In addition, the pooled DOR was 15.18 (95 % CI: 9.75–23.62, Fig. 4). For all above effect sizes, significant heterogeneities were observed (*P* < 0.001, *I*^*2*^ > 50 %). From the SROC in Fig. 4, AUC was 0.8666, and no threshold effect was found based on the Spearman correlation analysis (*P* = 0.616).

### Subgroup analysis

The results of subgroup analyses are summarized in Table 3. Higher sensitivity, specificity, PLR, DOR, AUC and Q*, and lower NLR were observed in the proven infection group (0.82, 0.74, 4.14, 30.58, 0.9136 and 0.8461, and 0.17) compared with those in clinical infection group (0.74, 0.66, 2.19, 6.98, 0.8245 and 0.7576, and 0.39). Slightly higher specificity, PLR and NLR, while lower sensitivity, DOR, AUC, and Q were found in the early-onset sepsis, compared with those in the late-onset sepsis. There were higher sensitivity, specificity, PLR and DOR, and lower NLR in term infants (0.80, 0.85, 5.75 and 24.07, and 0.24) compared with those in preterm infants (0.74, 0.69, 2.76 and 7.83, and 0.37).

**Table 3:** Subgroup analyses

Subgroup	Studies	Sensitivity (95 %)	Specificity (95 %)	PLR (95 %)	NLR (95 %)	SDOR (95 %)	AUC	Q*
All	21	0.77 (0.74, 0.79)	0.74 (0.72, 0.75)	3.58 (2.85, 4.49)	0.29 (0.22, 0.37)	15.18 (9.75, 23.62)	0.8666	0.7972
Infection								
Proven	7	0.82 (0.75, 0.87)	0.74 (0.71, 0.77)	4.14 (2.56, 6.68)	0.17 (0.06, 0.52)	30.58 (9.75, 95.88)	0.9136	0.8461
Clinical	5	0.74 (0.70, 0.77)	0.66 (0.62, 0.69)	2.19 (1.72, 2.79)	0.39 (0.25, 0.62)	6.98 (3.68, 13.24)	0.8245	0.7576
Clinical or Proven	9	0.78 (0.75, 0.81)	0.79 (0.77, 0.82)	3.93 (2.81, 5.49)	0.27 (0.21, 0.36)	15.41 (8.76, 27.09)	0.8661	0.7967
Type of sepsis								
Early-onset	7	0.75 (0.72, 0.78)	0.74 (0.72, 0.76)	3.57 (2.49, 5.11)	0.31 (0.23, 0.42)	12.40 (6.40, 24.00)	0.8415	0.7732
Late-onset	6	0.79 (0.75, 0.82)	0.71 (0.68, 0.74)	3.42 (2.11, 5.55)	0.24 (0.14, 0.41)	18.86 (6.82, 52.16)	0.9262	0.8606
Early & late-onset	8	0.78 (0.72, 0.84)	0.79 (0.74, 0.84)	3.65 (2.93, 4.57)	0.26 (0.11, 0.59)	20.43 (12.31, 33.91)	0.8835	0.8140
Infants								
Preterm	8	0.74 (0.71, 0.77)	0.69 (0.67, 0.71)	2.76 (2.16, 3.54)	0.37 (0.30, 0.47)	7.83 (4.84, 12.68)	0.8088	0.7436
Term	2	0.80 (0.74, 0.85)	0.85 (0.82, 0.88)	5.75 (3.08, 10.72)	0.24 (0.18, 0.31)	24.07 (13.17, 44.01)	--	--
Preterm + term	11	0.81 (0.76, 0.84)	0.81 (0.78, 0.84)	3.88 (3.04, 4.97)	0.21 (0.11, 0.41)	22.84 (12.94, 40.29)	0.8853	0.8159

### Meta-regression analysis

Meta-regression analysis (Table 4) showed that the “infants” was the cause of heterogeneity (*P* = 0.0147) and other variances were not the sources of heterogeneity (*P* > 0.05).

**Table 4:** Meta-regression

Variances	Coeff.	Std. Err.	*p* - value	RDOR	[95 % CI]
Cte.	1.002	0.729	0.1883	----	----
S	0.094	0.1956	0.6375	----	----
sepsis	-0.319	0.3179	0.3298	0.73	(0.37;1.43)
infants	0.806	0.2946	0.0147	2.24	(1.20;4.18)
diagnosis	0.436	0.2814	0.1406	1.55	(0.85;2.81)

## Discussion

nCD64 can be detected rapidly by flow cytometer with minimal blood volumes [6] and is reported widely to be used in the diagnosis of neonatal sepsis. This meta-analysis showed that the diagnostic performance of nCD64 for neonatal sepsis was not good, because the pooled sensitivity and specificity are not high enough. The PLR and NLR were also not satisfactory. Although the AUC is relatively high, the application of nCD64 for diagnosing neonatal sepsis needs to be cautious.

The pooled sensitivity and specificity of nCD64 were 77 % and 74 %, respectively, which are lower than those of serum procalcitonin (PCT) (81 % and 79 %), although AUC was similar (0.87) [31]. Indicators of nCD64 diagnostic value were lower than CRP (sensitivity 80.8 %, specificity 100 %, AUC 0.90), TNF-α (sensitivity 100 %, specificity 96.6 %, AUC 1) and IL-6 (sensitivity 96.2 %, specificity 89.7 %, AUC 0.97) according data of study of Kocabas E et al. [32]. Compared with the novel marker such as presepsin [33–37], nCD64 also showed a lower diagnostic efficiency. Thus, our results indicate that the nCD64 should not be used as a diagnostic marker alone for neonatal sepsis. It can be combined with other diagnostic methods like serum PCT [38] and hematologic scoring system (sensitivity 93 %; specificity 82 %) [39] to improve the diagnostic accuracy. The hematologic scoring system assigns one score for each of seven indexes (abnormal total leukocyte count, abnormal total neutrophil (PMN) count, elevated immature PMN count, elevated immature to total PMN ratio, Immature to mature PMN ratio ≥ 0.3, platelet count ≤ 150,000/mm^3^, and pronounced degenerative changes in PMNs) with higher scores indicating greater likelihood [39].

The results of the present study are similar with the previous meta-analysis of 12 studies (sensitivity, 78 %; specificity, 81 %; DOR, 21.27; PLR, 4.53; NLR, 0.23; AUC, 0.89.) [10]. Although nCD64 showed relatively high sensitivity and specificity in some included studies with cutoff of 2.3 % [7], 4000 phycoerythrinmolecules bound per cell [9], and 2.6 % [30], respectively, the small sample size and different cut-off may exaggerate the facticity of the results.

nCD64 expressed normally in non-infected neutrophils, but it could be up-regulated by stimulation of bacterial invasion [40]. It has been shown that the expression of nCD64 was not affected by transient tachypnea of the newborn (TTN), respiratory distress syndrome (RDS) and other non-infective perinatal events [21]. nCD64 expression in adults is different from newborn neonates. In adults, the expression of nCD64 may be higher in gram-negative sepsis than in gram-positive sepsis [41]. However, this difference has not been confirmed in neonates [21]. Neonates may have less expressed neutrophil to gram-negative bacteria infection. Furthermore, the expression of nCD64 may also been increased in leucocytes in patients with streptococcal infection [42]. All these lead to the lower power of nCD64 in diagnosis of neonatal sepsis.

Identification of the cut-off value of a diagnostic marker is difficult. If the cut-off value is high, the false positive rate may be overestimated. On the contrary, the low cut-off value may lead to overestimation of the false negative rate. Therefore, an appropriate cut-off value is necessary for improving the diagnostic accuracy of nCD64. In this study, cut-off values of nCD64 in included studies are different. Various cut-offs used in different studies might result in a threshold effect which is a cause of heterogeneity [43]. In the present study, no threshold effect was found based on the Spearman correlation analysis (*P* = 0.616), indicating that the threshold effect is not a cause of the high heterogeneity. The heterogeneity may be explained by the characteristics of the included patients. Some included neonates have other infections, which can also regulate the expression of nCD64. In addition, combination of studies with proven and clinical sepsis, data from preterm with term infants, and studies with early- and late-onset sepsis may also introduce heterogeneity. Therefore, we conducted the subgroup analysis based on these factors. The results revealed that higher sensitivity, specificity, PLR, AUC and Q* and lower NLR in the proven infection group than those in clinical infection group. There was higher sensitivity, specificity, PLR, DOR and lower NLR in term infants compared with those in preterm infants. No consistent differences in sensitivity, specificity, PLR, NLR, AUC and Q* were found between early-onset and late-onset sepsis. These results indicated that this method is more suitable for term infants than preterm infants, based on proven infection than other clinically suspected infection.

Heterogeneity is a common limitation of meta-analysis, especially in diagnostic meta-analysis. In the present study, meta-regression revealed that types of infants was one cause of the heterogeneity. Although subgroup analysis was performed based on the diagnostic method, types of sepsis (early-onset or late-onset), and preterm or term, the influences of other factors like the cutoff values were not assessed due to the lack of included studies and unavailable data. This reminds the clinical researchers providing more details of the patients in further studies, including the stage and types of neonatal sepsis. In addition, the appropriate and uniform cut-off value of nCD64 should be confirmed in further clinical studies.

## Conclusions

In conclusion, the n CD64 expression alone is not a satisfactory marker for diagnosing neonatal sepsis with relatively low sensitivity, specificity, PLR and NLR, in spite of relatively high SROC area. Therefore, the n CD64 expression used in diagnosis of neonatal sepsis should be treated with caution.   
